# Inter-enantiomer conversion dynamics and Johari–Goldstein relaxation of benzophenones

**DOI:** 10.1038/s41598-021-99606-0

**Published:** 2021-10-12

**Authors:** Michela Romanini, Roberto Macovez, Maria Barrio, Josep Lluís Tamarit

**Affiliations:** Grup de Caracterizació de Materials, Departament de Física, EEBE and Barcelona Research Center in Multiscale Science and Engineering, Universitat Politècnica de Catalunya, Av. Eduard Maristany 10-14, 08019 Barcelona, Catalonia Spain

**Keywords:** Chemistry, Physics

## Abstract

We employ temperature- and pressure-dependent dielectric spectroscopy, as well as differential scanning calorimetry, to characterize benzophenone and the singly-substituted ortho-bromobenzophenone derivative in the liquid and glass states, and analyze the results in terms of the molecular conformations reported for these molecules. Despite the significantly higher mass of the brominated derivative, its dynamic and calorimetric glass transition temperatures are only ten degrees higher than those of benzophenone. The kinetic fragility index of the halogenated molecule is lower than that of the parent compound, and is found to decrease with increasing pressure. By a detailed analysis of the dielectric loss spectra, we provide evidence for the existence of a Johari–Goldstein (JG) relaxation in both compounds, thus settling the controversy concerning the possible lack of a JG process in benzophenone and confirming the universality of this dielectric loss feature in molecular glass-formers. Both compounds also display an intramolecular relaxation, whose characteristic timescale appears to be correlated with that of the cooperative structural relaxation associated with the glass transition. The limited molecular flexibility of ortho-bromobenzophenone allows identifying the intramolecular relaxation as the inter-enantiomeric conversion between two isoenergetic conformers of opposite chirality, which only differ in the sign of the angle between the brominated aryl ring and the coplanar phenyl-ketone subunit. The observation by dielectric spectroscopy of a similar relaxation also in liquid benzophenone indicates that the inter-enantiomer conversion between the two isoenergetic helicoidal ground-state conformers of opposite chirality occurs via a transition state characterized by a coplanar phenyl-ketone moiety.

## Introduction

Molecular flexibility plays a crucial role for several important phenomena in the liquid state^1^, ranging from molecular self-assembly as in protein folding^2^, to the mechanical properties of synthetic polymers^3^, to the induced-fit model of biochemical reactions^4^. The degree of flexibility of an organic molecule is the result of more or less constrained internal rotations, that is, the ability of molecular moieties to rotate about covalent bonds within certain limits determined mainly by electronic configuration/conjugation and steric interactions. The simplest examples of constrained internal rotations are the torsional flexibility of ethane derivatives and linear homopolymers as polyethylene, or that of cyclic molecules, where internal C–C rotation is limited to few conformational states (e.g., the well-known chair or boat conformations of cyclohexane) by the closed-loop topology. Internal rotation is hindered by the presence of double bonds and of electronic resonances, e.g. in amide or phenyl groups. Even aromatic moieties, however, can rigidly rotate around exocyclic (peripheral) single C–C bonds. While the study of the molecular flexibility of large molecules is necessarily carried out, due to their complexity, on a case-by-case basis, it is important to identify and predict which types of constrained dynamics may take place in smaller subunits that are commonly found in larger organic molecules (e.g., the free rotation of methyl groups or the hindered rotation of phenyl groups)^5,6^.

Molecules may retain some of their internal degrees of freedom and thus a certain flexibility in the amorphous solid state and even in the crystalline state. The former case is interesting because, despite decades of intensive research, the glass transition and the kinetic stability of glasses remain open challenges of condensed matter science. The various theoretical approaches proposed, such as the mode-coupling theory or the coupling model, are only able to account for some aspects of this convoluted problem^7–10^. There are at least two difficulties inherent to any dynamic theory of glasses. The first difficulty is the several-orders of magnitude increase of the viscosity in a small temperature interval across the glass transition, and the simultaneous dramatic slow-down of the structural α relaxation, which is the main dynamic feature of glass-forming liquids and whose kinetic arrest marks the glass transition temperature *T*_g_. This feature is captured qualitatively by the Adam-Gibbs theory of 1965 and by its extension to polymers by Miller in 1978^11^, as well as by more quantitative and more recent theories^12^. The second difficulty is that real glassy systems exhibit elementary excitations on very different time scales, which range from the mHz frequency scale of the α relaxation near *T*_g_, through the radio and microwave region where so-called *secondary relaxations* are observed (some of which related to the molecular flexibility), up to the THz domain where an excess vibrational density of states (Boson peak) shows up^13–15^.

In the glass state, only secondary dynamic processes are active. Secondary relaxations are less cooperative relaxation modes than the structural α relaxation, and come in two varieties: non-diffusive, rigid molecular rototranslations known as Johari–Goldstein (JG) relaxations^16,17^, or internal rotations related to intramolecular (torsional) degrees of freedom. The JG secondary relaxations are believed to be intrinsic to the glass transition and to disordered systems in general^18^, and to play an important role for the kinetic stability of glasses^19^. In contrast, intramolecular relaxations do not seem to play a direct role in the glass transition, at least for small-molecule glass formers.

In the low frequency region (up to tens of GHz), supercooled isotropic liquids of rigid molecules exhibit only the α relaxation and a single JG relaxation (with the notable exception of methylindole, which exhibits two distinct rigid-rotation modes in the liquid state^20^). From a theoretical perspective, secondary relaxations that do not correspond to intramolecular degrees of freedom are predicted both by the Coupling Model^21,22^, according to which all glass formers should exhibit a “slow β relaxation” acting as “local precursor” of the α relaxation at higher frequency (shorter relaxation time), and by the mode-coupling theory^8,23^, which predicts the existence of a “fast β relaxation” at frequencies of hundreds of GHz, likely associated with the rattling motion of single molecules in the transient cage formed by its first neighbours. The two types of relaxations are observed in quite different frequency ranges and are considered independent relaxation processes. The existence of slow β relaxations, besides being a well-established experimental fact, is also predicted by numerical simulations of hard asymmetric molecules^24^, and by phenomenological modifications of the hard sphere model, e.g. the ENCLE model^25^.

**Figure 1:**
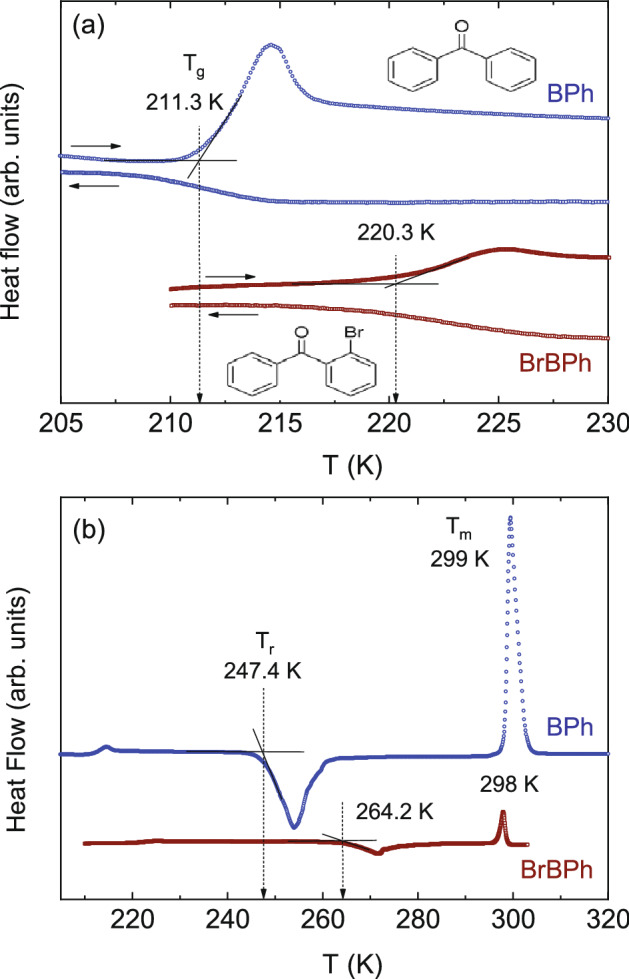
Ambient-pressure DSC thermograms (markers) of amorphous benzophenone (BPh) and ortho-bromobenzophenone (BrBPh) measured near the glass transition temperature (**a**) and upon heating to the melting point of the crystalline phase (**b**), normalized to sample mass. In (**a**), both cooling and heating ramps are shown, and the molecular structures of both compounds are displayed. In both panels an offset is introduced between the two traces for better clarity. Thin continuous lines indicate the determinations of the onset glass transition temperatures (*T*_g_) in (**a**) and the onset recrystallization temperatures (*T*_r_) in (**b**). Both are indicated by dashed vertical arrows. In (**b**), the melting points (peaks) are also indicated.

Most molecules are flexible, rather than rigid. In the liquid and glass states, they exhibit one or more secondary relaxations corresponding to internal degrees of freedom, which generally appear at higher characteristic relaxation frequency than the JG relaxation, if the latter is present. It has been reported that the JG relaxation is very weak or almost absent in the case of some flexible molecules^26^. Benzophenone (diphenylketone, see the inset to Fig. 1a for the two-dimensional molecular structure) is an interesting case because its secondary relaxations have been the object of controversy, and have raised the questions of whether a unified picture of both slow and fast β relaxations could be given^27^, and whether the slow β relaxation is really a universal feature of all glass formers^26^.

Benzophenone derivatives are an interesting class of very simple molecular compounds where both types of constraints on the rotation of phenyl groups, namely, steric interactions and electronic cross-conjugation, occur simultaneously. Cross-conjugation between the carbonyl and phenyl groups in benzophenone (BPh) leads to a helicoidal, symmetric “paddle-wheel” lowest-energy molecular conformation with the phenyl groups each rotated in opposite directions by roughly 30° with respect to the ketone plane (O=CR_2_, R=–C_6_H_5_). Two isoenergetic enantiomeric forms of opposite chirality exist. Liquid BPh displays a glass transition at *T*_g_ = 212 K^28^, and its structural (α) relaxation dynamics has been characterized by optical Kerr effect measurements^29,30^, depolarized light scattering^31^, and dielectric spectroscopy^27,32^. The latter studies have shown that it is a rather fragile glass-former, with kinetic fragility index^33,34^ of *m* = 125. Also the secondary relaxations of supercooled BPh have been studied with the same techniques. A fast secondary relaxation was first identified in optical studies conducted at 251 K, in the frequency range between 1 and 100 GHz. Later dielectric studies detected a temperature-dependent broad loss peak at the high-frequency side of the α relaxation.

The physical interpretation of these secondary processes has been the object of controversy. While the first studies of the optical Kerr effect in BPh reported^29,30^ that the results were in disagreement with the basic version of the mode-coupling theory, Götze and Sperl later showed that agreement could be recovered by extending the theory and that therefore the high-frequency secondary relaxation could be interpreted as the fast process predicted by the mode-coupling theory^23,35^. Lunkenheimer and coworkers showed that the secondary relaxation observed in dielectric experiments had a different origin than the fast relaxation^27^, and pointed^32^ to a possible JG character of the β relaxation. However, a dielectric spectroscopy characterization of amorphous BPh dispersions in a polymer matrix appeared almost at the same time has discarded the possibility that the dielectric β relaxation can be of JG origin, also due to the numerical disagreement between the experimental relaxation times and the predictions of the coupling model, without attempting an identification of this relaxation feature^36^.

In fact, identifying the exact molecular mechanism behind an intramolecular relaxation in BPh appears challenging. The two isoenergetic ground-state enantiomers of BPh are separated by a relatively low activation barrier^37,38^, with a conversion time of 12 ps at 333 K^39^. Since the conversion time becomes longer at lower temperature, this inter-enantiomer conversion might contribute to the relaxation spectrum. However, it cannot contribute to the dielectric response if it occurs via rotation only of the phenyl groups with respect to the central carbon atom, due to the constancy of the molecular dipole moment under such rotation.

In order to investigate the origin of the secondary dielectric response of BPh and further analyze the effect of constrained internal rotation in molecules with germinal aromatic rings, in the present contribution we analyze the temperature- and pressure-dependent relaxation dynamics of a singly-substituted halogenated derivative of BPh, namely ortho-bromobenzophenone (hereafter, BrBPh), and compare the results with more detailed dielectric spectra of BPh that we have acquired near the glass transition of this compound. The comparative analysis of the molecular relaxations of closely-related molecular derivatives can indeed be a valuable tool for the identification of secondary processes^40^. We choose BrBPh because it only differs from the parent molecule by substitution of a single atom, and it has better glass-forming ability and kinetic stability of the supercooled liquid phase than pristine BPh. A brominated derivative is chosen in particular because, compared to molecular species containing e.g. hydroxyl or amine functional groups, the intermolecular coupling and molecular dynamics of halogenated derivatives are expected to be simpler due to the lack of hydrogen-bond interactions, so that self-aggregation and networking are not expected to occur, which enables a simpler identification of relaxation processes^41–44^.

Both BPh and BrBPh possess geminal aryl groups covalently linked to a central carbonyl group. The brominated derivative differs only in the presence of a bromine atom at the 2-position of one of the phenyl rings (insets to Fig. 1a). This substitution gives rise, however, to a strong molecular asymmetry, accompanied by (i) a more pronounced steric hindrance of the halogenated aryl group compared to the phenyl groups of the parent molecule, and (ii) a strongly asymmetric molecular and electronic structure. In fact, while as mentioned BPh adopts a helicoidal structure favouring cross-conjugation between the central carbonyl group and the two equivalent phenyl moieties, in BrBPh the electronegativity of the Br substituent leads to strong π-electron delocalization only between the carbonyl group and the non-substituted phenyl ring, which results in a planar arrangement of the phenyl-ketone moiety, as evidenced by a recent crystal structure study of both solid-state polymorphs of BrBPh^45^.

We first provide an assignment of the relaxation modes of BrBPh, and then compare the results with new measurements that we perform on BPh near its glass transition. We show that both BPh and BrBPh display a JG relaxation whose spectral position matches that predicted by the Coupling Model, and an intramolecular relaxation. These findings confirm the existence of a JG relaxation also in the controversial BPh case. Since the BrBPh molecule possesses essentially only one degree of freedom, its intramolecular relaxation can be ascribed to the interconversion between two isoenergetic BrBPh enantiomers of opposite chirality, which involves a reorientation of the brominated ring with respect to the coplanar phenyl-ketone moiety. Interestingly, this intramolecular relaxation scales with the structural relaxation time when the pressure is varied. We argue that the intramolecular relaxation of BPh similarly involves a transition state with one of the phenyl rings coplanar with the ketone group, that is, a structure analogous to the lowest-energy conformation of the brominated derivative. With this assignment, we are able to provide a complete picture of the relaxation dynamics of both molecular liquids.

## Experimental details

Benzophenone (BPh, molecular formula C_13_H_10_O) was purchased from Acros Organics (Thermo Fisher) with purity better than 99%. Ortho-bromobenzophenone (BrBPh, molecular formula C_13_H_9_BrO) was purchased from Aldrich with a purity better than 98%. Both compounds were used as received. Calorimetric experiments were carried out on both compounds with a commercial Q100 thermal analyzer from TA Instruments (New Castle), connected in the case of BPh to a liquid N_2_ dewar, on samples of the order of 10 mg sealed in aluminum pans. Details of the temperature and enthalpy calibration of the device have been given elsewhere^46^. The glass transition and recrystallization (cold crystallization) temperatures were determined from the onset of the corresponding calorimetric features.

Complex permittivity spectra of supercooled liquid BrBPh and BPh were acquired by means of broadband dielectric spectroscopy, using mainly a Novocontrol analyzer working in the frequency range from 10^−2^ to 10^7^ Hz and connected to a capacitor containing the sample, in set-ups allowing control of temperature (and in the case of BrBPh, also pressure). Additional ambient-pressure high-frequency dielectric spectroscopy measurements were carried out on BrBPh by means of an HP4291 impedance analyzer working in the frequency range from 10^6^ to 1.8·10^9^ Hz in reflectometry geometry, with the sample capacitor mounted at the end of a coaxial cable. The liquid phase was obtained by heating the as-received powders above their respective melting points directly inside stainless-steel parallel-plate capacitors of two different types, designed specifically to carry out experiments on liquids both at ambient pressure and under an applied hydrostatic pressure. In all experiments, the electrodes of the parallel-plate capacitors were kept at fixed distance by means of silica spacers of 50 μm diameter.

Ambient-pressure dielectric experiments were conducted on both compounds by placing the capacitors in a Novocontrol Quatro temperature controller. High-pressure experiments on BrBPh were carried out using a thermal bath (Lauda Proline RP 1290) with a liquid-flow circuit connected to a high-pressure setup. Both setups provided temperature control with an error not higher than ± 0.3 K. For the measurements at high pressure, the capacitor was covered by a teflon membrane and latex wrapping to prevent a possible contamination with the pressurizing fluid (thermal oil from Huber). The insulated capacitor was then placed in a high-pressure chamber (Unipress) made of a Cu-Be alloy, which was filled with the thermal oil and connected to a manual pump that allowed applying hydrostatic pressure between ambient pressure and 0.6 GPa, as determined with a pressure transducer with 0.5% accuracy.

Dielectric spectra of BrBPh were acquired at fixed (*T*, *P*) conditions. Ambient-pressure data were acquired both while increasing and lowering the temperature in steps of 2 or 4 K, while high-pressure spectra were collected only while lowering the temperature, because the sample underwent recrystallization (see “Results and discussion” section) during the step-wise temperature ramp required for the dielectric measurement. In the BPh case, dielectric spectra were acquired only at ambient pressure at temperature close to the glass transition of this compound, since at higher temperature or pressure the supercooled liquid phase underwent recrystallization. In comparison with previous work^27^, we used a larger frequency interval and smaller frequency factor between consecutive points in the spectra, to be able to better identify the number of loss components.

We analyzed all acquired loss spectra (imaginary part of the complex permittivity) by modeling each relaxation process as the imaginary part of the phenomenological Havrilak-Negami (HN) equation^47^:

1 $$\varepsilon^{*} \left( \omega \right) = \varepsilon_{\infty } + \frac{\Delta \varepsilon }{{\left( {1 + \left( {i\omega \tau_{HN} } \right)^{a} } \right)^{b} }}.$$

Here ω = 2π*f* is the angular frequency, ε_∞_ is the real part of the permittivity on the high-frequency side of the loss feature, Δε is the dielectric intensity (strength), and *a* and *b* are shape parameters. From the fit parameter τ_HN_, the characteristic relaxation time τ_max_ corresponding to the maximum dielectric loss was determined as:

2 $$\tau_{max} = \tau_{HN} \left[ {\sin \left( {\frac{a\pi }{{2b + 2}}} \right)} \right]^{ - 1/a} \left[ {\sin \left( {\frac{ab\pi }{{2b + 2}}} \right)} \right]^{1/a}$$

After performing several fits of spectra acquired at different (*T*, *P*) values we concluded that for both compounds, the best fit for the primary (α) relaxation was obtained for *a* = 1, a special case of the general HN function known as Cole-Davidson function. The secondary relaxations were instead modeled assuming *b* = 1, in which case the HN function reduces to the Cole–Cole function and τ_max_ coincides with the fit parameter τ_HN_.

The same fit functions (Cole-Davidson for the primary α relaxation, Cole–Cole for the secondary relaxation(s)) were employed in previous dielectric studies of BPh^27,32^. In contrast with previous studies on BPh, however, we have modeled all our spectra as the sum of three relaxation features (structural relaxation plus two secondary relaxations). The need for a three-component model for the loss spectra is discussed in detail in “Results and discussion” section. The so-called “dynamic” glass transition temperature was determined, as customary, as the temperature at which the primary relaxation time τ_α_ (τ_max_ in Eq. 2) reached 100 s.

## Results and discussion

Figure 1 displays the DSC traces of amorphous BPh and BrBPh, acquired upon heating from the glass state obtained by cooling from the liquid phase at a rate of 10 K/min. The typical signature of the glass transition is visible at 211.3 K and 220.3 K in the pristine and halogenated molecules, respectively (Fig. 1a). The two glass transitions temperatures (*T*_g_) are comparable to those reported in previous studies^28,45^. The slightly higher glass transition temperature in BrBPh, which has higher molecular weight M_w_, is in qualitative agreement with the reported general correlation between *T*_g_ and M_w_ in van-der-Waals molecular liquids, albeit the expected *T*_g_ for BrBPh should be 15% higher if the empirical rule^48^ *T*_g_ ~ M_w_^1/2^ were fulfilled ((M_w,BrBPh_/M_w, BPh_)^1/2^ = (261.1/182.2)^1/2^ ≅ 1.2 < T_g,BrBPh_/T_g,BPh_ = 1.05). Despite the large molecular mass difference, also the melting points of the two compounds coincide roughly (Fig. 1b).

**Figure 2:**
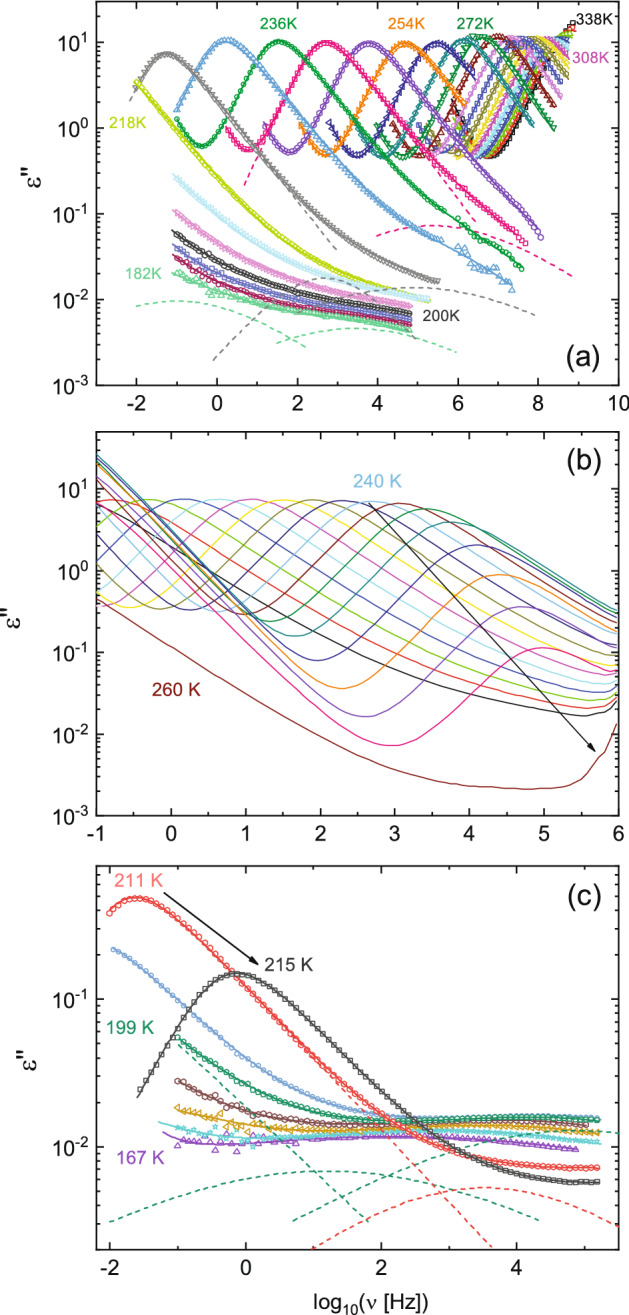
Ambient-pressure isothermal dielectric loss spectra of both studied compounds. (**a**, **b**) Spectra of BrBPh at selected temperatures between 182 and 338 K, acquired both upon lowering the temperature (**a**) and upon increasing it (**b**). (**c**) Spectra of BPh acquired while increasing the temperature from well within the glass state. In (**a**) and (**c**), data are represented as markers, continuous lines are fits, and dotted lines are fit components, which are shown only for few representative spectra, namely at 182, 224 and 242 K for BrBPh (**a**) and 199 and 211 K for BPh (**c**). In both (**b**) and (**c**), recrystallization is visible as a marked decrease of the intensity of the main loss (α relaxation) feature above a certain temperature (*T* > 240 K for BrBPh (**b**), *T* > 211 K for BPh (**c**)).

Both supercooled liquids displayed a strong tendency to recrystallize, as visible in the thermograms where the recrystallization (cold crystallization) exotherms can be discerned with onset temperatures *T*_r_ ≈ 247 K for BPh and *T*_r_ ≈ 264 K for BrBPh, respectively (Fig. 1b). Recrystallization of both compounds was observed also in the series of dielectric spectra acquired with an increasing temperature ladder at ambient pressure, where the onset of recrystallization took place at even lower temperatures (just above 240 K for BrBPh, Fig. 2b, and just above *T*_g_ for BPh, Fig. 2c). For this reason, in order to characterize the relaxation features of BrBPh, most isothermal dielectric permittivity spectra on this compound were acquired using a descending temperature program, both at ambient pressure (Fig. 2a) and under an applied hydrostatic pressure. This procedure indeed prevented recrystallization, likely by avoiding the nucleation of seeds, which generally occurs at lower temperature than the optimum growth rate^49,50^. For similar reasons, we limited our measurements on BPh to temperatures close to *T*_g_ at ambient pressure, as liquid BPh always crystallized in our set-up upon application of a hydrostatic pressure.

Figure 2a displays representative isothermal loss spectra of the cooling-down series at ambient pressure, in the range between 338 and 182 K (spectra were acquired every 2 K but are displayed every 6 K). The spectra are dominated by a relaxation process at low frequency, visible as a broad asymmetric maximum of the imaginary permittivity. Just above the calorimetric glass transition temperature (220 K), the peak of this main loss feature is observed between 0.01 and 0.1 Hz, which allows identifying it as the structural α relaxation of BrBPh. Similarly, the loss spectra of BPh (Fig. 2c) exhibited a main α relaxation loss feature whose kinetic freezing marks the glass transition temperature of this compound, in agreement with previous studies^27,32^. Both compounds exhibited an excess loss intensity on the high-frequency flank of the α peak, indicative of the presence of one or more secondary relaxations.

**Figure 3:**
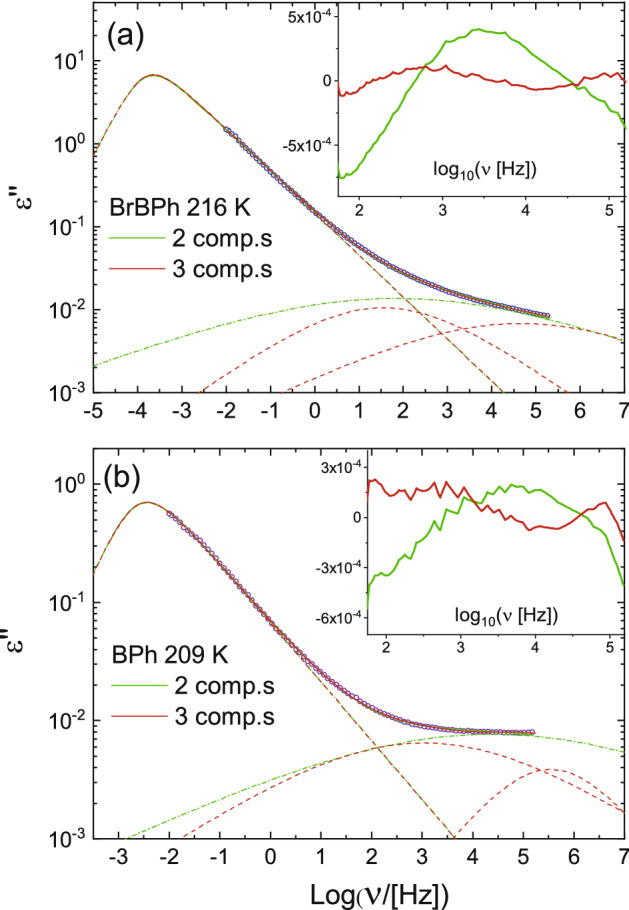
Comparison between the two-component and three-component fits of the ambient-pressure loss spectrum of BrBPh at *T* = 216 K (**a**) and of BPh at *T* = 209 K (**b**). Markers are data points, continuous lines are fits, and dashed lines represent fit components. The relative difference between either fit and the experimental spectrum are displayed in the inset to each panel.

In order to identify the number of (secondary) component present in the spectra, and extract their characteristic relaxation time, we applied the fitting procedure described in “Experimental details” section. Figure 3 compares fits of the loss spectrum of BrBPh at 216 K (a) and that of BPh at 209 K (b) with either two or three relaxation components. These temperatures were close to the respective glass transition temperature of each compound. The mean-square deviation of the fit with three components was only slightly lower than that with only two. However, the difference between the spectra and both fits, displayed in the insets, significantly shows a systematic, non-random frequency dependence of the fit error in the case of only two loss components (green curves), which displayed a peak-like feature at frequencies intermediate between the two components.

This result is clearly indicative of the presence, in both compounds, of an intermediate relaxation between the main α relaxation and the loss component at highest frequency. This is in disagreement with previous studies^27,32^ which reported only two relaxation processes (namely, the main α relaxation and the highest-frequency one) in pristine BPh. The failure of previous studies to identify the intermediate loss process is probably due to the fact that this earlier analysis was based on spectra containing fewer data points (see “Experimental details” section), possibly to avoid crystallization of BPh during the dielectric measurements.

**Figure 4:**
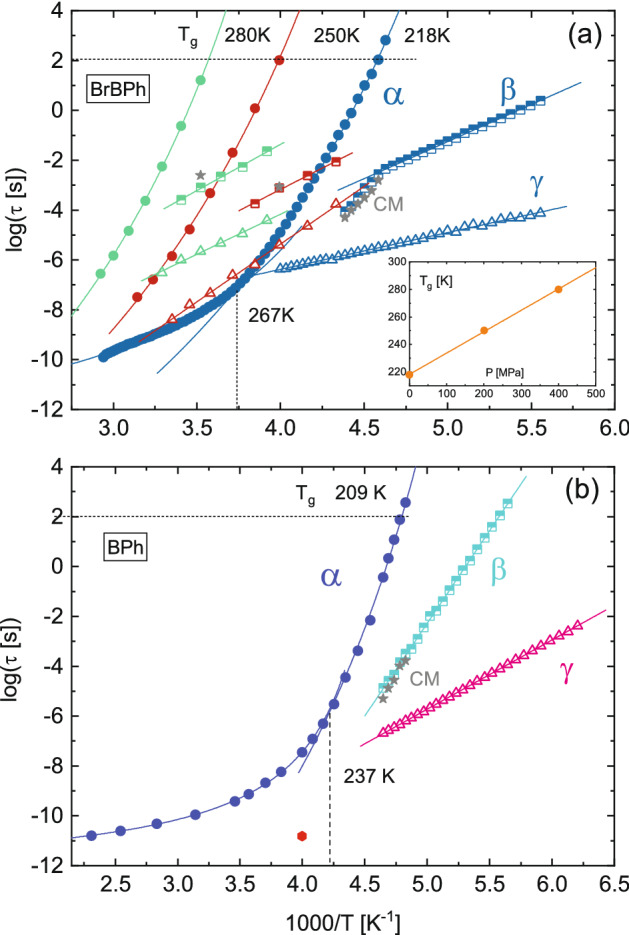
Arrhenius plot of the structural (α, filled circles) and secondary β (half-open squares) and γ (open triangles) relaxation times in BrBPh (**a**) and BPh (**b**). In (**a**), the data for all three studied pressures are reported (blue: 0.1 MPa; red, 201 MPa; green: 400 MPa). In (**b**), α relaxation times above *T*_g_ were obtained by our own fits of the loss spectra of Ref.^27^, and the red dot represents the relaxation time of the fast-β relaxation inferred from analysis of the optical Kerr effect on BPh (Ref.^35^). In both panels, continuous lines are VFT fits (Eq. 3) of the α relaxations or linear (Arrhenius law) fits of the secondary (β, γ) relaxation times. The dashed horizontal line marks the glass transition temperature *T*_g_, while the dashed vertical line marks the dynamic cross-over temperature at which the VFT temperature dependence of the α relaxation changes in the ambient pressure data. Stars represents the precursor times predicted by the Coupling Model (Eq. 5). Inset to (**a**): variation of *T*_g_ with the applied pressure (markers). The line is a linear fit.

Three-component fits were carried out on the loss spectra of both compounds at all measured (*P*, *T*) conditions. As mentioned in “Experimental details” section, the α relaxation was well described by a Cole-Davidson function, which is a special case of the HN Eq. (1) with *a* = 1, while both secondary relaxations were modeled as Cole–Cole functions, a special case of the HN Eq. (1) with *b* = 1. A Cole-Davidson function for the α relaxation was employed also in previous dielectric studies on BPh^27,32^, while a Cole–Cole line shape is a usual feature of secondary relaxations^51^. The relaxation times τ (Eq. 2) of each relaxation are displayed as a function of inverse temperature (Arrhenius plot) in Fig. 4. As customary, the three processes are labeled as α, β, and γ starting from the one visible at lowest frequency (longest characteristic time τ). It can be observed that all three relaxations become slower (display longer τ) as the temperature is lowered or as the pressure is increased, as expected for molecular relaxation processes.

The time–temperature Arrhenius relaxation map of amorphous BrBPh is displayed in Fig. 4a for the three pressures studied, namely ambient pressure (0.1 MPa), 201, and 400 MPa. For the α relaxation, the observed effect of pressurization entails that the kinetic glass transition temperature (see below) becomes larger with increasing hydrostatic pressure, as expected. Moreover, the behavior of the structural relaxation time with temperature and pressure is compatible with the expected decrease of the effective activation volume of the relaxation with increasing temperature^52^.

The α relaxation displays super-Arrhenius behaviour at all three studied pressures. At least close to the glass transition, this behaviour could be well modelled with the Vogel-Fulcher-Tamman (VFT) function, given by^53–55^:

3 $$\tau_{\alpha } \left( T \right) = { }\tau_{\infty } {\text{exp}}\left( {D\frac{{T_{VF} }}{{T - T_{VF} }}} \right)$$

Here τ_∞_ is the characteristic time extrapolated at very high (infinite) temperature, *D* is the so-called fragility strength coefficient and *T*_VF_ is the Vogel-Fulcher temperature. It should be noted that, for the ambient-pressure data of BrBPh, a single VFT equation was not sufficient to fit all structural relaxation times. Instead, two VFT equations had to be employed for both compounds, one in the temperature range close to the glass transition, and a different one for the data at higher temperatures. The same behaviour was reported previously for the structural relaxation of BPh^27,32^.

The so-called dynamic glass transition temperature *T*_g_ was determined from the intersection between the lower-temperature VFT fit of the α relaxation and the line of equation Log(τ/[s]) = 2 (horizontal dashed lines in Fig. 4). In both cases, the dynamic *T*_g_ at ambient pressure (209 and 218 K for the pristine and brominated compounds, respectively) was only slightly lower (by 2 K) than the calorimetric one (211 and 220 K). Since *T*_g_ is not a thermodynamically defined but rather a kinetically defined temperature, a small discrepancy can be expected due to the different experimental conditions: the calorimetric *T*_g_ is measured in the heat-up ramp of a cool-heat cycle at 10 K/min, while the dynamic (dielectric) *T*_g_ is determined in the present case upon cooling or heating slowly in a stepwise fashion during acquisition of the isothermal loss spectra. Since the acquisition of a dielectric permittivity spectrum down to frequencies of 10^–2^ Hz takes roughly one hour, the experimental procedure allows the supercooled liquid to retain its ergodic state at lower temperature compared with the faster DSC temperature ramp, which rationalizes why *T*_g,BDS_ < *T*_g,DSC_.

The (*T*_g_,*P*_g_) pairs extracted from the dielectric measurements of BrBPh at different pressures are plotted in the inset to Fig. 4a. It can be seen that *T*_g_ scales linearly with *P*_g_ in the studied pressure range. This linearity most likely holds only at low enough applied pressures; indeed, studies performed in a larger pressure interval usually show a non-linear dependence, often described by the Anderson-Anderson equation^56,57^. The numerical values of *T*_g_ and *P*_g_ are reported in Table 1 along with the VFT fit parameters (only one set of parameters are reported for the ambient pressure data, namely those corresponding to the VFT closer to *T*_g_). Table 1 also includes the isobaric kinetic fragility index, which is defined as^58^:

4 $$m_{P} = \left[ {\frac{d}{{d\left( {T_{g} /T} \right)}}\log \left( {\tau_{\alpha } } \right)} \right]_{{T = T_{g} }}$$

**Table 1:** Dynamic glass transition temperatures, VFT parameters of the primary α relaxation in the proximity of *T*_g_, and kinetic fragility index of BrBPh and BPh.

Compound	Ortho-bromobenzophenone			Benzophenone
*P*_g_ (MPa)	0.1	201	400	0.1
*T*_g_ (K)	218	250	280	209
Log(τ_∞_/[s])	− 21.5 ± 0.5	− 21.3 ± 1.4	− 20.5 ± 0.9	− 23.6 ± 0.3
*T*_VF_ (K)	141.1 ± 2.4	154.8 ± 9.6	180.5 ± 6.4	143.6 ± 0.5
D	29.7 ± 2.0	33.2 ± 7.3	28.7 ± 3.9	26.6 ± 2.6
m_*P*_	63 ± 10	56 ± 10	54 ± 10	82 ± 10
m_*P*, VFT_	68 ± 10	62 ± 10	64 ± 10	81 ± 10

The latter was computed either from a linear fit (m_*P*_) or from the VFT parameters (m_*P*,VFT_).

The numerical value of *m*_*P*_ was determined either directly from a linear fit to the experimental data near *T*_g_, or from the VFT fit parameters describing the structural relaxation near the glass transition (denoted as *m*_*P*, VFT_); both values are reported in Table 1. The isobaric fragility of BrBPh is smaller than that of the parent BPh molecule, and it appears to decrease slightly with increasing pressure. The fragility index of BPh is smaller than that reported in a previous study^27^.

In contrast to the structural relaxation, the secondary relaxations of both compounds exhibited a simply-activated Arrhenius behaviour (straight-line appearance in the relaxation maps of Fig. 4) both at ambient and high pressure (for BrBPh). While the γ relaxation was characterized by a single activation energy in all cases, the β relaxation of BrBPh displayed a cross-over between a lower activation barrier in the glass state (below *T*_g_) and a higher one in the supercooled liquid state (above *T*_g_), at least in the spectra acquired at ambient pressure (Fig. 4a). This cross-over in the temperature dependence at *T*_g_ is typical of the Johari–Goldstein (JG) relaxations^18^ mentioned in the introduction.

According to the Coupling Model^59,60^, the JG relaxation time near *T*_g_ should match approximately the so-called precursor time *τ*_CM_, given by:

5 $$\tau_{{{\text{CM}}}} = t_{c}^{1 - k} \left( {\tau_{\alpha } } \right)^{k} .$$

Here *t*_c_ ≈ 2 · 10^–12^ s is a cut-off time universal to molecular and polymeric glass formers^18^, τ_α_ is the structural relaxation time, and *k* is the exponent of the stretched Kohlrausch–Williams–Watts exponential function describing the α loss feature in the time domain. For most cases^61,62^, the latter quantity can be accurately estimated from the *a* and *b* exponents of the HN Eq. (1) of the α relaxation as $k \cong \left( {ab} \right)^{{\frac{1}{1.23}}}$.

Near the dynamic *T*_g_ at ambient pressure, the *a* and *b* HN fit parameters of the α relaxation of BrBPh had typical values of 1 (Cole-Davidson function) and 0.58, respectively, leading to a Kohlrausch exponent *k* of 0.64. A similar Kohlraush exponent was found for the high pressure spectra of BrBPh. The corresponding *k* value for unsubstituted BPh at ambient pressure was somewhat lower, 0.57. As it may be observed in Fig. 4, the calculated precursor times (stars) for both supercooled liquid BrBPh and BPh just above *T*_g_ are virtually identical to the experimental β relaxation times both at ambient and high pressure.

**Figure 5:**
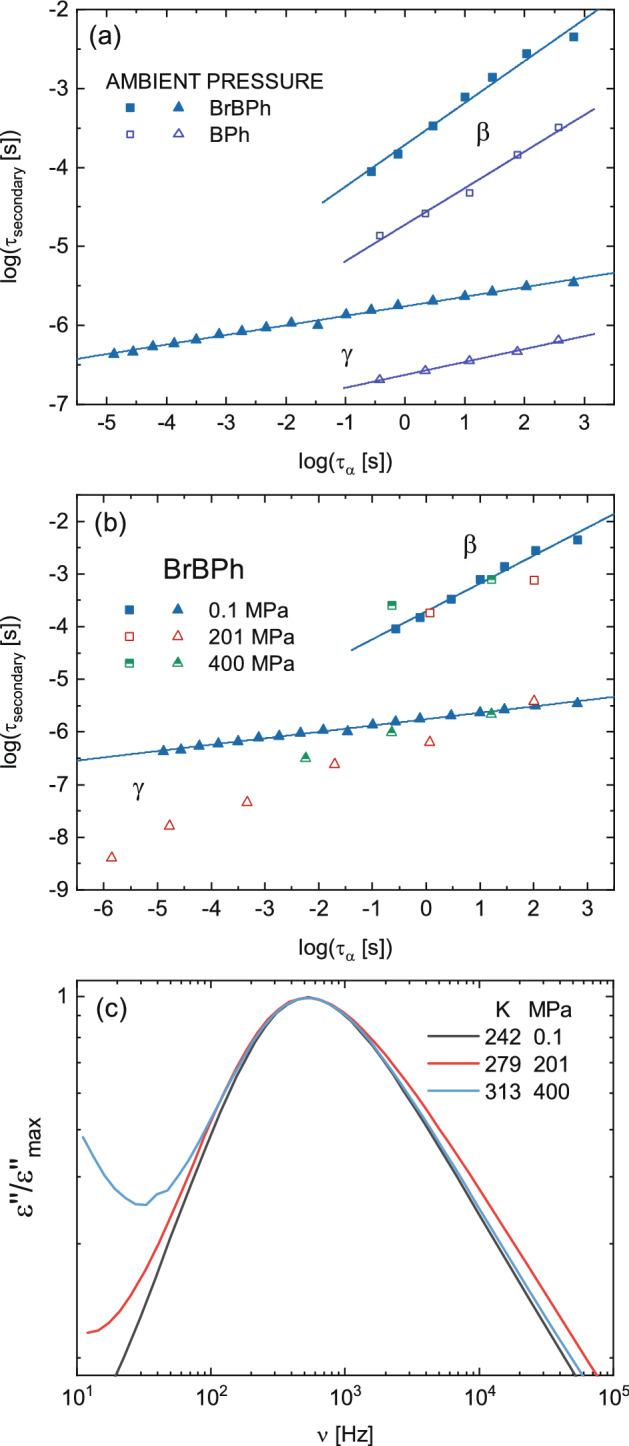
(**a**) Logarithmic plot of τ_β_ and τ_γ_ as a function of τ_α_ (above *T*_g_) for the ambient pressure data of BrBPh (filled markers) and BPh (open markers), and linear fits. (**b**) Same plot for BrBPh for all three measured pressures. Continuous lines are linear fits of the ambient pressure data. (**c**) Normalized isochronal plot of the loss spectra of BrBPh at three (*T*, *P*) pairs for which the structural relaxation frequency is roughly the same (namely, τ_α_ ≅ 3 · 10^–4^ s).

To further corroborate the JG nature of the β relaxation of both compounds, Fig. 5 displays the logarithmic plot of both τ_β_ and τ_γ_ as a function of τ_α_, for all acquired data. At ambient pressure (Fig. 5a), the logarithm of both secondary relaxation times is linearly correlated with log(τ_α_), with slopes of 0.53 ± 0.03 and 0.12 ± 0.01 for τ_β_ and τ_γ_, respectively, for BrBPh, and 0.46 ± 0.03 and 0.16 ± 0.01 for BPh. According to Eq. (5), in such plot the JG relaxation time should exhibit a slope equal to the Kohlrausch exponent *k*. The experimental slopes of log(τ_β_) vs log(τ_α_) are only slightly higher than the experimental value of *k* in both compounds, confirming the JG nature of this relaxation. In contrast, in both cases the slopes of the logarithmic τ_γ_ *vs* τ_α_ plots are way too small to correspond to a JG relaxation. Indeed, such low slope value would entail, according to Eq. (5), an extremely broad α relaxation feature of both compounds, which is not observed in the loss spectra (see Fig. 2b and c). This is in agreement with the conclusions of Ref.^36^ that the γ relaxation of BPh cannot be a JG process. It can be further observed in Fig. 5b that the high-pressure data for τ_β_ for BrBPh fall roughly on top of the corresponding ambient pressure data, which is to be expected for a JG relaxation if the Kohlrausch exponent is not sensitive to pressure. This is instead not observed for the γ relaxation of the brominated derivative. A further visual proof of this common scaling of τ_β_ and τ_α_ can be obtained by comparing isochronal spectra, that is, spectra at distinct (*T*, *P*) pairs with a common structural relaxation time. Three such isochronal spectra, with very similar relaxation time τ_α_ ≅ 3 · 10^–4^ s, are compared in Fig. 5c. The spectra are normalized to the maximum loss of the structural relaxation, and they are slightly shifted in frequency for better visual comparison. It can be seen that the shape of the loss spectra is roughly invariant, suggesting that the spectral (frequency) position of the β relaxation is the same in all isochronal spectra despite the relatively large change in (*P*, *T*) values. Such spectral invariance is a feature of JG relaxations.

**Figure 6:**
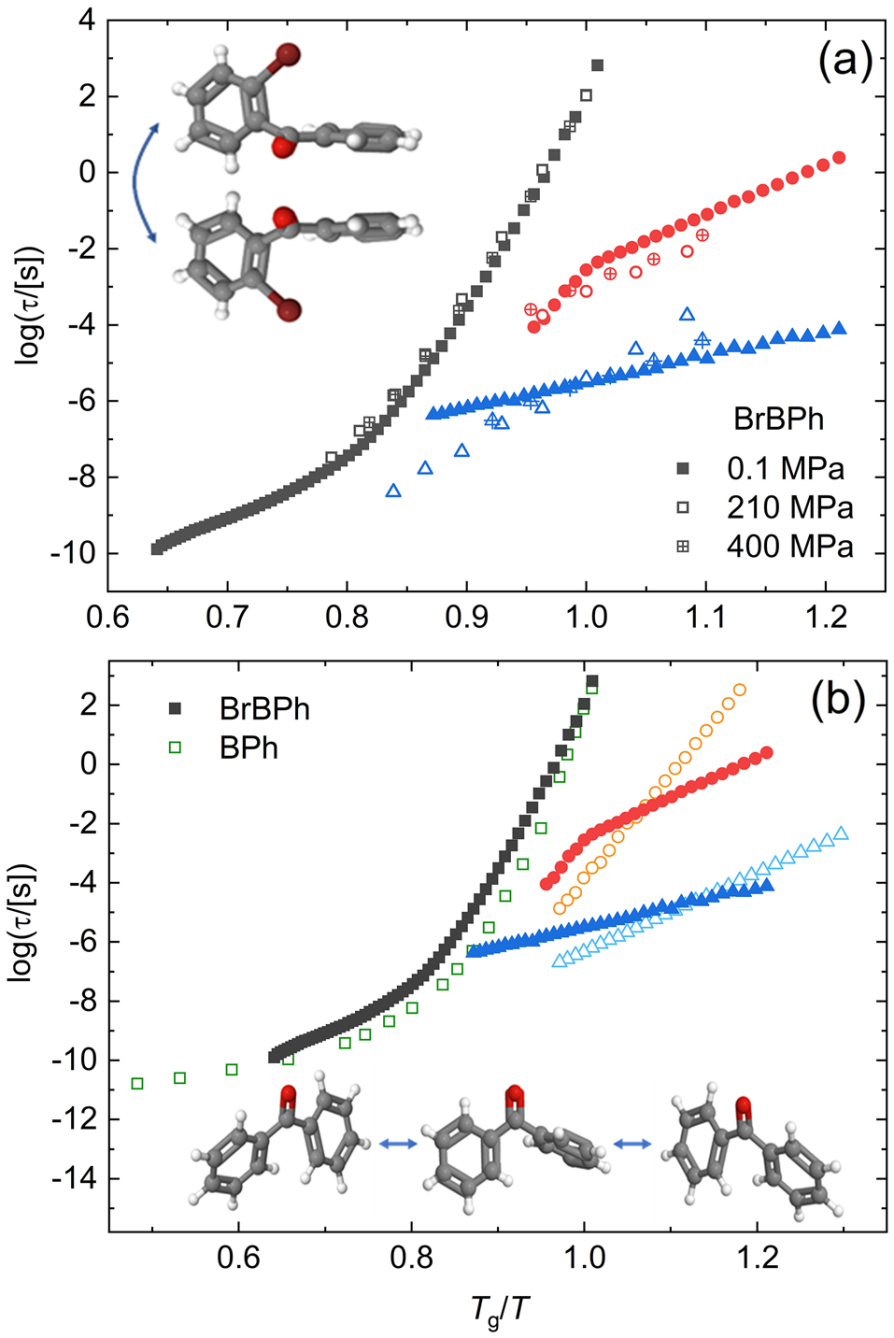
(**a**) Angell plot of all dielectric relaxation times of BrBPh at various (*T*,*P*) conditions (same data as in Fig. 4). Inset: proposed conformational change of the BrBPh molecules, corresponding to the γ relaxation. (**b**) Ambient-pressure comparative Angell plots of BrBPh and BPh. Inset: proposed pathway of the conformational change of the BPh molecules, corresponding to the γ relaxation of this compound.

The correlation between the β relaxation and the structural one can be also discerned in the so-called Angell plot, the plot of the logarithm of the relaxation times as a function of the inverse reduced temperature, defined as the temperature normalized to the glass transition temperature at a given pressure. The Angell plot of all BrBPh data is displayed in Fig. 6a. It can be observed that the logarithmic relaxation times of the JG β relaxation scale roughly with *T*_g_ and that they display the same low-temperature slope in the Angell plot, regardless of the applied pressure. The slope of the γ relaxation is instead different with varying pressure. The application of a hydrostatic pressure has been sometimes employed to distinguish the JG relaxation from intramolecular processes^57^, since the JG relaxation is expected to display a master-curve scaling in the Angell plot for different pressures, while intramolecular relaxations are not. This test could not be performed for supercooled BPh due to its strong tendency to crystallize under an applied pressure.

For comparison purposes, we plot in Fig. 6b the ambient-pressure data for both BPh and BrBPh in a single Angell plot. The more pronounced curvature of the α relaxation of pristine BPh in the Angell plot is consistent with its higher kinetic fragility index (Eq. 4). The β JG data of the two compounds are not superposed in the common Angell plot, which is not surprising considering that the precursor time of the Coupling Model depends not only on τ_α_ but also on the shape parametres of the α relaxation.

Having assigned the slowest (α) and intermediate (β) dielectric relaxations of both compounds to the structural and Johari–Goldstein relaxation processes, respectively, the fastest relaxation (γ) can only have an intramolecular character. Indeed, the molecule is not flat as methylindole, the only known example of a rigid molecule with two secondary whole-molecule relaxations in the isotropic liquid state^20^.

As mentioned in the introduction, a recent X-ray diffraction study of the two crystalline forms of BrBPh has shown that the molecular structure of the brominated derivative is not helicoidal as the ground-state structure of unsubstituted BPh, but rather displays a coplanar phenyl-ketone moiety, tilted with respect to the brominated ring^45^. This molecular geometry entails the existence of two possible enantiomers with same energy and opposite chirality, and both the stable and metastable crystal phases of BrBPh are in fact racemic mixtures with equal population of both enantiomers^40^. Since the metastable crystalline phase is formed by recrystallization from supercooled liquid BrBPh, it is likely that the inter-enantiomer conversion is active in the liquid state (we cannot exclude a priori the possibility that conformers with different internal torsion angles coexist in the liquid phase). We therefore assign the γ relaxation of BrBPh to an inter-enantiomer (or inter-conformer) conversion dynamics in the liquid phase. Such internal rotation dynamics is detected by dielectric spectroscopy because both the brominated ring and the phenyl-ketone coplanar moiety have non-zero electric dipole moment.

One possibility for the γ relaxation of BrBPh is that it corresponds to the chirality inversion dynamics between the two isoenergetic equilibrium enantiomers (see the inset to Fig. 6a). It should be remarked that, due to steric repulsion between the Br atom on one hand, and the carbonyl oxygen and the hydrogen groups of the phenyl ring on the other hand, such chirality inversion cannot take place simply by a rigid relative rotation of the brominated ring with respect to the phenyl-ketone moiety, but must involve a transition state in which the phenyl ring and the carbonyl group are no longer coplanar and the phenyl-ketone cross-conjugation is thus partially removed. In other words, the same steric repulsion between the bromine atom and the hydrogen atoms of the phenyl ring which forbids the fully planar configuration of BrBPh (and in general of molecules with two germinal aryl groups), also prevents inter-enantiomer conversion via a pure torsional motion of the planar phenyl-ketone moiety with respect to the brominated ring. Another possibility for the γ relaxation could be a small-angle rigid relative reorientation of the two planar subunits of the BrBPh ground-state conformer, without a change in chirality. As we discuss in the next paragraphs, the comparison with BPh suggests that the first interpretation is the most likely one.

It is noteworthy that log(τ_γ_) and log(τ_α_) are linearly correlated in both compounds (Fig. 5). Since according to the Debye-Stokes–Einstein and Maxwell relations the structural relaxation time is proportional to the viscosity, the correlation between τ_γ_ and τ_α_ in BrBPh (Fig. 5b) implies that the internal rotation of BrBPh is a power-law function of the viscosity. Intramolecular relaxations are generally found to be independent of the structural mobility and of the viscosity^63,64^, especially when they correspond to a torsional degree of freedom involving only a small subunit of a larger molecule. In the case of BrBPh, however, the inter-enantiomeric conversion involves the relative reorientation of two moieties that constitute the whole of the molecule. Such reorientational motion is necessarily hindered by intermolecular steric interactions, especially if an inter-enantiomer conversion takes place, which may rationalize the observed correlation with the structural mobility and viscosity. Given both the large mass of bromine (basically equal to the mass of the whole phenyl group, so that the brominated ring has roughly twice the phenyl mass) and the significantly larger volume and steric hindrance of the brominated ring compared to the pristine phenyl ring, it is likely that the γ relaxation actually involves the internal and external rotation of the (more mobile) phenyl-ketone moiety, with respect to a brominated ring that remains stationary.

The assignment of the γ dielectric process in pristine BPh is less straightforward. In fact, the γ relaxation of BPh cannot correspond to a simple rotation of one or both phenyl rings, because such rotation would not result in a variation of the magnitude or direction of a dipole moment, other than a possible small effect due to cross-conjugation resonance when the phenyl ring becomes coplanar with the carbonyl group. As a consequence, a rotation of only the phenyl groups cannot be detected in dielectric spectroscopy.

Although neither the β nor the γ relaxations are superposed in Fig. 6b, they occur in the same reduced temperature-frequency sector of the Angell plot in both compounds. This similarity between the two compounds and their Angell plots, the observation that the intramolecular γ relaxation scales with the structural mobility also in BPh (Fig. 5a), and the fact that γ relaxation must involve a reorientation of the carbonyl group of the BPh species, all suggest that the γ relaxation of BPh corresponds to a an inter-enantiomer or inter-conformer conversion dynamics similar to that of BrBPh.

It has been shown in a gas-phase theoretical study^37^ that, although the coexistence of rotational conformers of BPh can be ruled out, the conformation of BPh with a phenyl ring coplanar to the carbonyl group corresponds to a saddle point in the rotational energy landscape which represents the minimum-energy interconversion path between the two lowest-energy helicoidal paddle-wheel enantiomers. Physically, this minimum energy path is due to the fact that the fully planar phenyl-ketone moiety has maximum cross-conjugation between the central carbonyl and one of the phenyl rings, and also due to the fact that it avoids strong steric interactions between the phenyl rings during their relative rotation. In view of this, we tentatively assign the γ relaxation of BPh to an interconversion process involving a transition state with the same configuration as BrBPh, namely, with one of the phenyl rings coplanar with the ketone group and the other one tilted (see the inset to Fig. 6b for a visual scheme of such a process), and ascribe the γ relaxation of BrBPh to the chirality inversion process.

With this assignment, the observation of a dielectrically-active inter-conformer dynamics in BPh can be accounted for. Just like the inter-enantiomer conversion process of BPh occurs through a “perpendicular” transition state^37^, due to similar steric and electronic interactions the inter-enantiomer conversion process of BrBPh occurs through a conformation in which both conjugated rings are out-of-plane, similarly to the helicoidal conformation of the ground state BPh conformer. In this sense, therefore, *the proposed assignment of the γ relaxations of both pristine and brominated benzophenone involves precisely the same conformations*, in one case as equilibrium states, and in the other case as transition state*.* In the transition state^37^ of BPh (respectively, equilibrium state of BrBPh^45^), electronic conjugation between the coplanar phenyl and carbonyl moieties is maximum. On the other hand, the equilibrium helicoidal conformation of BPh has maximum cross-conjugation between the phenyl group (only partial cross-conjugation between both phenyl rings can be obtained, since the coplanar configuration of both rings is forbidden by steric interaction). As mentioned in the introduction this effect is less important in BrBPh due to the asymmetry caused by the electronegativity of the Br atom.

We therefore suggest that steric and electronic conjugation effects establish a preference for these two types of conformers during dynamic inter-enantiomer conversion processes in both compounds. The molecular weight of the brominated aryl ring is more than twice that of the unsubstituted phenyl ring, and the molecular volume occupied by the brominated ring is correspondingly larger, so that we can expect that the γ relaxation time is determined by the same inertial effects/steric interactions that are responsible for the higher glass transition temperature of the brominated derivative. This helps rationalize the scaling of both γ relaxations with the corresponding τ_α_ times.

## Conclusions

We have employed temperature- and pressure-dependent dielectric spectroscopy to characterize the molecular dynamics of the supercooled liquid and glassy states of benzophenone and ortho-bromobenzophenone. The dielectric loss spectra of both compounds exhibit three separate relaxation processes, visible in distinct temperature/frequency ranges, which are assigned either to rigid rototranslations (α, β) or to internal rotations (γ). The relaxation at lowest frequency correspond to the structural α dynamics whose kinetic freezing marks the glass transition temperature *T*_g_, which at ambient pressure takes place at 209 and 218 K in the pristine and brominated compounds, respectively. In the halogenated compound, the *T*_g_ is observed to scale linearly with applied pressure.

The intermediate-frequency relaxation in both compounds corresponds to the non-diffusive, whole-molecule Johari–Goldstein (JG) β secondary relaxation, as determined both by comparison with the Coupling Model and, in the case of the halogenated derivative, by the common scaling with applied hydrostatic pressure. The observation of such relaxation also in pristine benzophenone resolves a long-standing issue about the possible lack of the JG relaxation in this molecule.

The third and highest-frequency γ relaxation corresponds to an intramolecular process, which in ortho-bromobenzophenone stems from the only internal molecular degree of freedom, namely, the external rotation of the brominated ring with respect to the coplanar, cross-conjugated phenyl-ketone moiety. Due to steric interactions between the hydrogen and bromine groups of the geminal aryl rings, the planarity of the carbonyl-phenyl moiety has to be broken temporarily for such internal rotation to take place. We assign the γ relaxation of otho-bromobenzophenone to the interconversion between two isoenergetic enantiomers of opposite chirality. The observation of a dielectrically active γ relaxation also in the parent compound can be rationalized by invoking an internal rotation of the molecule that involves a change of orientation of the carbonyl moiety, which is the only polar group of the benzophenone molecule. We therefore suggest that the γ relaxation of benzophenone stems from the intramolecular dynamics involving a transition state with the same geometry as the brominated derivative, namely, with the phenyl ring coplanar with the carbonyl group. Our contribution shows that the comparative analysis of the relaxation map of related molecular derivatives is a valuable tool for the experimental identification of molecular dynamics processes.

## References

[1] Sung W (2018). Statistical Physics for Biological Matter.

[2] Lindorff-Larsen K, Piana S, Dror RO, Shaw DE (2011). How Fast-Folding Proteins Fold. Science.

[3] Doi M, Edwards SF (1978). Dynamics of concentrated polymer systems. Part 1. Brownian motion in the equilibrium state. J. Chem. Soc. Faraday Trans..

[4] Otten R (2020). How directed evolution reshapes the energy landscape in an enzyme to boost catalysis. Science.

[5] Eliel EL, Wilen SH, Mander LN (1994). Stereochemistry Of Organic Compounds.

[6] Dunbrack RL, Cohen FE (1997). Bayesian statistical analysis of protein side-chain rotamer preferences. Protein Sci..

[7] Donth E (2001). The Glass Transition.

[8] Götze W, Sjogren L (1992). Relaxation processes in supercooled liquids. Rep. Prog. Phys..

[9] Tanaka H (1996). A self-consistent phase diagram for supercooled water. Nature.

[10] Kivelson SA, Tarjus G (2008). In search of a theory of supercooled liquids. Nat. Mater..

[11] Larson RG (1999). The Structure and Rheology of Complex Fluids.

[12] Mirigian S, Schweizer KS (2013). Unified theory of activated relaxation in liquids over 14 decades in time. J. Phys. Chem. Lett..

[13] Zaccone A (2020). Relaxation and vibrational properties in metal alloys and other disordered systems. J. Phys. Condens. Matter.

[14] Cummins HZ (2005). Dynamics of supercooled liquids: Excess wings, β peaks, and rotation translation coupling. J. Phys. Condensed Matter.

[15] Berthier L, Charbonneau P, Jin Y, Parisi G, Seoane B, Zamponi F (2016). Growing timescales and lengthscales characterizing vibrations of amorphous solids. Proc. Nat. Acad. Sci..

[16] Johari GP, Goldstein M (1970). Viscous liquids and the glass transition. II. Secondary relaxations in glasses of rigid molecules. J. Chem. Phys..

[17] Johari GP, Goldstein M (1970). Molecular mobility in simple glasses. J. Chem. Phys..

[18] Ngai KL, Paluch M (2004). Classification of secondary relaxation in glass-formers based on dynamic properties. J. Chem. Phys..

[19] Kissi EO, Grohganz H, Löbmann K, Ruggiero MT, Zeitler JA, Rades T (2018). Glass-transition temperature of the β-relaxation as the major predictive parameter for recrystallization of neat amorphous drugs. J. Phys. Chem. B.

[20] Tu W, Valenti S, Ngai KL, Capaccioli S, Liu YD, Wang L-M (2017). Direct evidence of relaxation anisotropy resolved by high pressure in a rigid and planar glass former. J. Phys. Chem. Lett..

[21] Ngai KL, Rizos AK, Plazek DJ (1998). Reduction of the glass temperature of thin freely standing polymer films caused by the decrease of the coupling parameter in the coupling model. J. Non-Cryst. Solids.

[22] Rajagopal AK, Ngai KL, Teitler S (1991). Theoretical aspects of coupling model schemes of slow relaxation in complex correlated systems. J. Non-Cryst. Solids.

[23] Götze W, Sperl M (2004). Nearly logarithmic decay of correlations in glass-forming liquids. Phys. Rev. Lett..

[24] Fragiadakis D, Roland CM (2013). Characteristics of the Johari-Goldstein process in rigid asymmetric molecules. Phys. Rev. E.

[25] Phan AD, Thuy TTT, An NTK, Knapik-Kowalczuk J, Paluch M, Wakabayashi K (2020). Molecular relaxations in supercooled liquid and glassy states of amorphous gambogic acid: Dielectric spectroscopy, calorimetry, and theoretical approach. AIP Adv..

[26] Shahin Thayyil M, Capaccioli S, Prevosto D, Ngai KL (2008). Is the Johari-Goldstein β-relaxation universal?. Philos. Mag..

[27] Pardo LC, Lunkenheimer P, Loidl A (2007). Dielectric spectroscopy in benzophenone: The β relaxation and its relation to the mode-coupling Cole-Cole peak. Phys. Rev. E.

[28] Davydova, N. A., Melnik, V. I., Nelipovitch, K. I. & Baran, J. Low-frequency Raman scattering from glassy and supercooled liquid benzophenone. *J. Mol. Struct.* **563–564**, 105 (2001)

[29] Cang H, Novikov VN, Fayer MD (2003). Experimental observation of a nearly logarithmic decay of the orientational correlation function in supercooled liquids on the picosecond-to-nanosecond time scales. Phys. Rev. Lett..

[30] Cang H, Novikov VN, Fayer MD (2003). Logarithmic decay of the orientational correlation function in supercooled liquids on the Ps to Ns time scale. J. Chem. Phys..

[31] Brodin A, Rössler EA (2006). Depolarized light scattering versus optical Kerr effect spectroscopy of supercooled liquids: Comparative analysis. J. Chem. Phys..

[32] Lunkenheimer P, Pardo LC, Köhler M, Loidl A (2008). Broadband dielectric spectroscopy on benzophenone: α relaxation, β relaxation, and mode coupling theory. Phys. Rev. E.

[33] Plazek DJ, Ngai KL (1991). Correlation of polymer segmental chain dynamics with temperature-dependent time-scale shifts. Macromolecules.

[34] Böhmer R, Angell CA (1992). Correlations of the nonexponentiality and state dependence of mechanical relaxations with bond connectivity in Ge-As-Se supercooled liquids. Phys. Rev. B.

[35] Sperl M (2006). Cole-Cole law for critical dynamics in glass-forming liquids. Phys. Rev. E.

[36] Capaccioli S, Shahin Thayyil M, Ngai KL (2008). Critical issues of current research on the dynamics leading to glass transition. J. Phys. Chem. B.

[37] Baraldi I, Gallinella E, Momicchioli F (1986). Conformations and internal rotation properties of molecules containing one geminal diphenylgroup: diphenylethylene, diphenylketimine, benzophenone, diphenylether and diphenylmethane. J. Chim. Phys..

[38] Cough KM, Wildman TA (1990). Hindered internal rotation in benzophenone. J. Am. Chem. Soc..

[39] Di Carlo EN, Smyth CP (1962). Microwave absorption and molecular structure in liquids. XLVIII. The dielectric relaxation of diphenyl sulfide, triphenylamine and diphenylmethane. J. Am. Chem. Soc..

[40] Valenti S, Barrio M, Negrier P, Romanini M, Macovez R, Tamarit JL (2021). Comparative physical study of three pharmaceutically active benzodiazepine derivatives: Crystalline versus amorphous state and crystallization tendency. Mol. Pharmaceut..

[41] Elton DC (2017). The origin of the Debye relaxation in liquid water and fitting the high frequency excess response. Phys. Chem. Chem. Phys..

[42] Gabriel J, Pabst F, Helbling A, Böhmer T, Blochowicz T (2018). Nature of the debye-process in monohydroxy alcohols: 5-Methyl-2-hexanol investigated by depolarized light scattering and dielectric spectroscopy. Phys. Rev. Lett..

[43] Romanini M, Mitsari E, Tripathi P, Serra P, Zuriaga M, Tamarit JL, Macovez R (2018). Simultaneous orientational and conformational molecular dynamics in solid 1,1,2-trichloroethane. J. Phys. Chem. C.

[44] Romanini M, Barrio M, Macovez R, Capaccioli S, Tamarit JL (2019). Mixtures of m-fluoroaniline with apolar aromatic molecules: Phase behaviour, suppression of H-bonded clusters, and local H-bond relaxation dynamics. J. Mol. Liquids.

[45] Romanini M (2021). Uniaxial negative thermal expansion in polymorphic 2-bromobenzophenone, due to aromatic interactions?. Cryst. Growth Des..

[46] Barrio M, Espeau P, Tamarit JL, Perrin M-A, Veglio N, Ceolin R (2009). Polymorphism of progesterone: relative stabilities of the orthorhombic phases I and II inferred from topological and experimental pressure-temperature phase diagrams. J. Pharm. Sci..

[47] Havriliak S, Negami S (1967). A complex plane representation of dielectric and mechanical relaxation processes in some polymers. Polymer.

[48] Novikov VN, Rössler EA (2013). Correlation between glass transition temperature and molecular mass in non-polymeric and polymer glass formers. Polymer.

[49] Carpentier L, Desprez S, Descamps M (2003). Crystallization and glass properties of pentitols. J. Therm. Anal. Calorim..

[50] Descamps M, Dudognon E (2014). Crystallization from the amorphous state: Nucleation-growth decoupling, polymorphism interplay, and the role of interfaces. J. Pharm. Sci..

[51] Kremer F, Schönhals A (2003). Broadband Dielectric Spectroscopy.

[52] Mpoukouvalas K, Floudas G, Williams G (2009). Origin of the α, β, (βα), and “slow” dielectric processes in poly(ethyl methacrylate). Macromolecules.

[53] Fulcher GS (1925). Analysis of recent measurements of the viscosity of glasses. J. Am. Ceramic Soc..

[54] Tammann G, Hesse W (1926). Die Abhängigkeit der Viscosität von der Temperatur bie unterkühlten Flüssigkeiten. Z. Anorg. Allg. Chem..

[55] Vogel H (1921). Das temperaturabhängigkeitsgesetz der viskosität von flüssigkeiten. Phys. Z..

[56] Floudas G, Paluch M, Grzybowski A, Ngai K (2011). Molecular Dynamics of Glass-Forming Systems. Effects of Pressure; Advances in Dielectric.

[57] Romanini M, Barrio M, Macovez R, Ruiz-Martin MD, Capaccioli S, Tamarit JL (2018). Thermodynamic scaling of the dynamics of a strongly hydrogen-bonded glass-former. Sci. Rep..

[58] Böhmer R, Ngai KL, Angell C, Plazek D (1993). Nonexponential relaxations in strong and fragile glass formers. J. Chem. Phys..

[59] Ngai KL (1998). Relation between some secondary relaxations and the α relaxations in glass-forming materials according to the coupling model. J. Chem. Phys..

[60] Ngai KL (2007). Why the glass transition problem remains unsolved?. J. Non-Cryst. Solids.

[61] Alvarez F, Alegría A, Colmenero J (1991). Relationship between the time-domain Kohlrausch-Williams-Watts and frequency-domain Havriliak-Negami relaxation functions. Phys. Rev. B.

[62] Alvarez F, Alegría A, Colmenero J (1993). Interconnection between frequency-domain Havriliak-Negami and time-domain Kohlrausch-Williams-Watts relaxation functions. Phys. Rev. B.

[63] Romanini M (2018). Enhancement of the physical and chemical stability of amorphous drug-polymer mixtures via cryogenic comilling. Macromolecules.

[64] Valenti S, Diaz A, Romanini M, del Valle LJ, Puiggali J, Tamarit JL, Macovez R (2019). Amorphous binary dispersions of chloramphenicol in enantiomeric pure and racemic poly-lactic acid: Morphology, molecular relaxations, and controlled drug release. Int. J. Pharmaceut..

